# Tomato ripeness and stem recognition based on improved YOLOX

**DOI:** 10.1038/s41598-024-84869-0

**Published:** 2025-01-14

**Authors:** Yanwen Li, Juxia Li, Lei Luo, Lingqi Wang, Qingyu Zhi

**Affiliations:** College of Information Science and Engineering, Shanxi Agricultural University, Jinzhong, 030800 China

**Keywords:** Recognition of tomato maturity, Deep learning, Fruit stem recognition, Attention module, Loss function, Computational biology and bioinformatics, Image processing

## Abstract

To address the challenges of unbalanced class labels with varying maturity levels of tomato fruits and low recognition accuracy for both fruits and stems in intelligent harvesting, we propose the YOLOX-SE-GIoU model for identifying tomato fruit maturity and stems. The SE focus module was incorporated into YOLOX to improve the identification accuracy, addressing the imbalance in the number of tomato fruits and stems. Additionally, we optimized the loss function to GIoU loss to minimize discrepancies across different scales of fruits and stems. The mean average precision (mAP) of the improved YOLOX-SE-GIoU model reaches 92.17%. Compared to YOLOv4, YOLOv5, YOLOv7, and YOLOX models, the improved model shows an improvement of 1.17–22.21%. The average precision (AP) for unbalanced semi-ripe tomatoes increased by 1.68–26.66%, while the AP for stems increased by 3.78–45.03%. Experimental results demonstrate that the YOLOX-SE-GIoU model exhibits superior overall recognition performance for unbalanced and scale-variant samples compared to the original model and other models in the same series. It effectively reduces false and missed detections during tomato harvesting, improving the identification accuracy of tomato fruits and stems. The findings of this work provide a technical foundation for developing advanced fruit harvesting techniques.

## Introduction

In tomato intelligent harvesting operations, the harvesting of different maturity fruits is important to ensure fruit quality and reduce storage costs^1^. Among these, recognizing fruit ripeness and stalks is key to the practical implementation of intelligent harvesting. Identifying different maturity of tomato fruits is a necessary prerequisite for the realization of fine intelligent harvesting. At present, many researchers apply machine vision technology to the intelligent tomato harvesting, with a particular focus on the accurate identification detection and positioning of tomato^2,3^

Traditional machine vision technology integrates image processing with machine learning techniques. However, the method is affected by background information when detecting unripe tomatoes and fruit stalks in complex background images. Furthermore, the process of manually extracting features is required. Additionally, the method exhibits limited generalization ability, so it is difficult to obtain better detection results^4–6^. Compared to the previously described method, deep learning requires only a labelled tomato dataset, from which the features of the target can be extracted without the need for human design of features^7–9^. This approach is becoming increasingly prevalent in the field of agricultural target detection. Among commonly used target detection networks, the network of YOLO (You Only Look Once) series is more streamlined than other networks, runs the fastest, has better real-time performance, and has a wide range of applications^10–13^. Li Tianhua et al.^14^combined the HSV method in conjunction with YOLOv4 to segment the red region of tomatoes, facilitating the recognition of ripening tomato in a complex environment. He Bin et al.^15^ proposed a YOLOv5 network based on the CIoU loss function, which achieved 96.2% and 97.6% of mAP for green and red fruits of tomato in the nighttime environment, and in this study, we proposed a model to recognize tomato ripening in greenhouse based on the improved YOLOv5 tomato ripeness recognition model,  YOLOv5s-tomato. Cheng Wei et al.^16^ recognized tomato red and green fruits in a greenhouse based on the improved YOLOv3 model, achieving an average recognition detection accuracy of 95.7%. Yang Jian et al.^17^ introduced a method for recognizing tomato ripeness using an enhanced YOLOv4-tiny model. To enhance the recognition accuracy of obscured tomatoes, they incorporated the Convolutional Block Attention Module (CBAM) into the backbone of the YOLOv4-tiny model. Taiheng Z et al.^18^ improved YOLOv5 model through the implementation of lightweight modifications and the utilization of a genetic algorithm for the optimization of hyperparameters, thereby enhancing the detection accuracy to a mAP of 96.9%. Fang Liu et al.^19^ developed an enhanced multi-scale YOLO algorithm designed to extract more feature information, thereby improving the speed and accuracy of tomato fruit detection. Liu et al.^20^ introduced YOLO-Tomato, an advanced tomato detection model based on YOLOv3. This model adopts a circular bounding box for tomato localization instead of the traditional rectangular one. Lv et al.^21^ used an improved combined augmented YOLOX-ViT model to collaboratively recognize tomato flowers and fruits in the greenhouse, achieving an average accuracy of 92.30%. Li et al.^22^ based their research on an improved YOLOv5, proposing the tomato maturity recognition model YOLOv5s-tomato, which uses EIoU loss to replace the original loss function, achieving an average accuracy of 97.42%. Lawal^23^ improved the precise identification of tomatoes in complex environments by applying labeling method, dense structure merging, spatial pyramid pooling and the Mish activation function within the enhanced YOLOv3 model.

The majority of the studies referenced above have focused on the identification of red and green tomato fruits. However, in the process of harvesting tomatoes, it is essential to accurately distinguish between different maturity levels to satisfy the requirements of transportation and storage. However, due to the brief period of fruit maturation, the class labels are imbalanced, and issues such as the differences of stalk size and their tendency to be obscured make it difficult to distinguish different maturity tomatoes and fruit stalks at the same time. To address these issues, this experiment proposes the YOLOX-SE-GIoU model, which adds the SE attention module to the original YOLOX model and optimizes the loss function to GIoU loss. This achieves precise recognition of tomatoes at different maturities and peduncles in greenhouse environments.

## Materials and methods

### Data collection

**Fig. 1:**
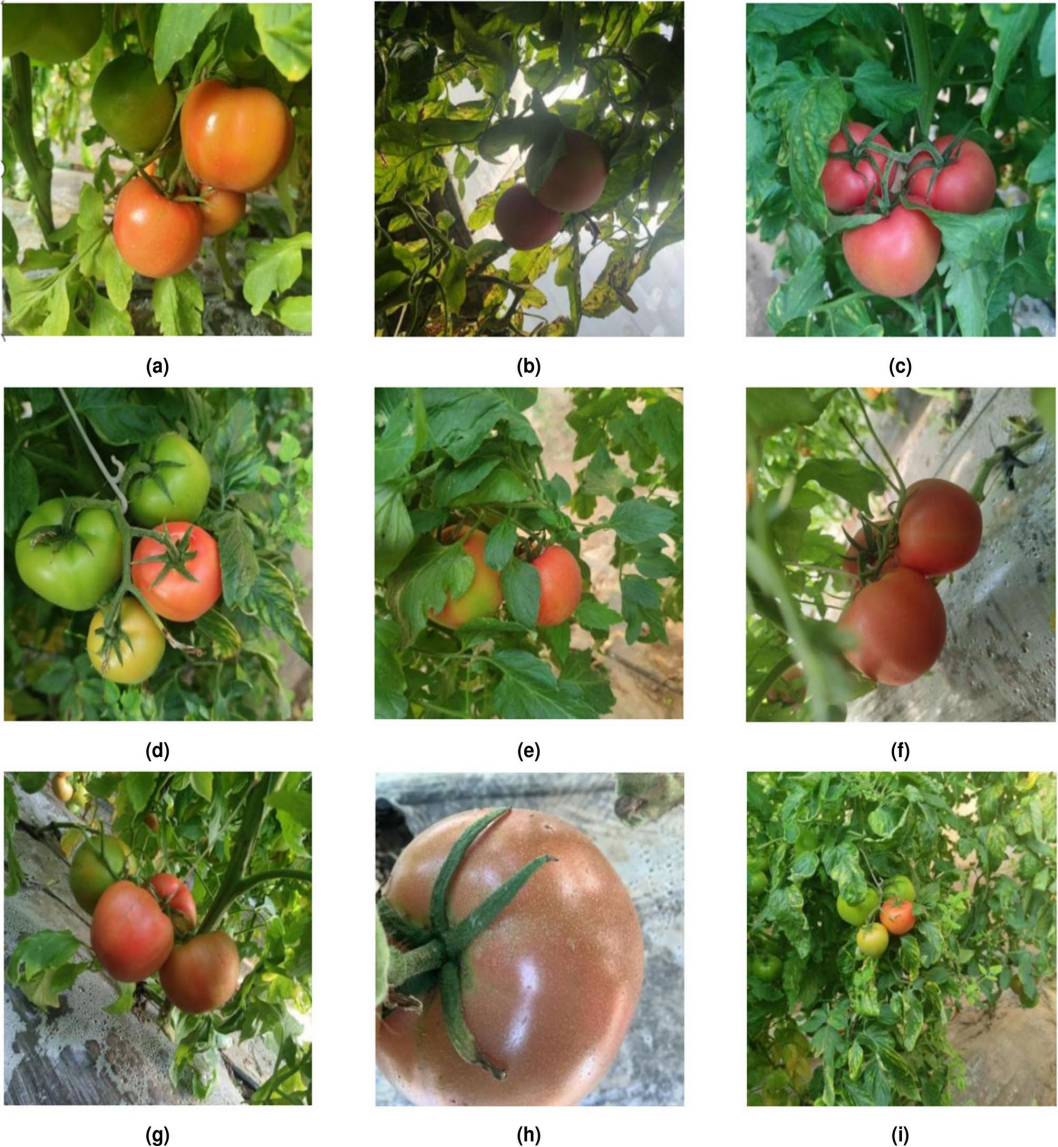
Tomato sample data in complex environments. (**a**) Image of tomatoes under direct sunlight; (**b**) Image of tomatoes in a backlit environment; (**c**) Image of a tomato on a overcast; (**d**) Image of unobscured tomato; (**e**) Image of tomato obscured by leaves; (**f**) Image of tomato obscured by branches; (**g**) Image of overlapping tomato fruits; (**h**) Image of tomato taken at close range; (**i**) Image of tomatoes taken at a distance.

The RGB tomato image dataset used in this experiment was collected in November 2022 from the entrepreneurial park of Shanxi Agricultural University, Taigu District, Shanxi Province, and the tomato variety was Provence. The image data was collected using an Apple iPhone 14 Pro smartphone. The image resolution was set to 4624 × 4624 pixels to ensure high-quality image acquisition and data reliability. To ensure the diversity of tomato fruit and fruit stalk identification in complex environments and the authenticity of the planting scene, different time periods (7:00 to 22:00) and different shooting angles(horizontal and overhead) were used to collect different ripeness, different light, different degrees of shading, different distances (maintaining a linear distance of 300-550 mm between the camera and the tomato fruits), and different numbers of fruit were collected in a total of 1,300 tomato images. The data samples for the tomatoes in different scenes are shown in Fig. 1.

### Datasets construction

The current national standard GH/T1193-2021 classifies tomato maturity into six categories: unripe, green ripe, color-changing, pre-red ripe, mid-red ripe, and post-red ripe based on color and size. The morphological characteristics of tomato fruits of different maturity levels are shown in Table 1.

**Table 1:** Morphological characteristics of tomato fruits with different ripeness.

Maturity level	Morphological characteristics
Unripe	The fruit and seeds have not yet fully grown and developed into their final form. The fruit skin is green and dull. Ripening is difficult. They are unsuitable for harvesting and storage.
Green ripe	The fruit has taken shape, the surface is shiny, transitioning from green to white-green. Seeds have grown larger and are surrounded by a gelatinous substance. At this stage, artificial ripening, harvesting, and storage are feasible.
Color-changing	This is the transition period from green ripeness to red ripeness. Yellow or light red spots begin to appear around the fruit’s navel. Less than 10% of the fruit surface shows red coloration.
Pre-red ripe	10% to 30% red ripeness: 10% to 30% of the fruit surface shows red coloration.
Mid-red ripe	40% to 60% red ripeness: 40% to 60% of the fruit surface shows red coloration.
Post-red ripe	70% to 100% red ripeness: 70% to 100% of the fruit surface shows red coloration.

The collected images were filtered according to the standard of fruit morphological characteristics, and 1,000 images were selected to construct the dataset for this experiment. The training and testing sets were randomly divided in an 8:2 ratio, with 20% of the training set allocated as the validation set for cross-validation during model training. Consequently, the training set consisted of 640 images, the test set included 200 images, and the validation set contained 160 images.

**Fig. 2:**
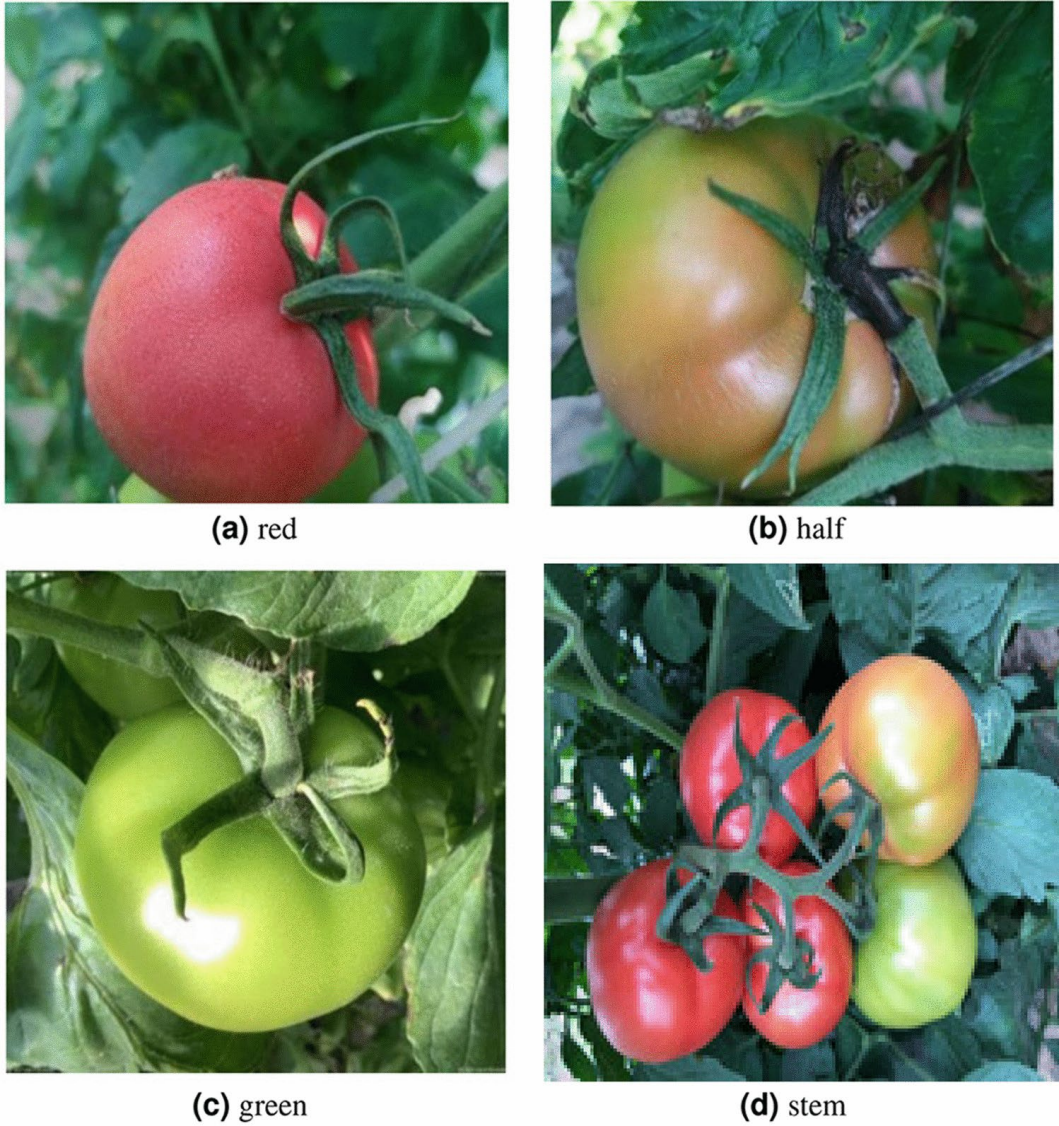
Sample of tomato maturity classification.

Due to the presence of multiple maturity levels within a single image, strictly adhering to these standards became highly complex. To simplify the classification process and improve model training efficiency, tomato fruits and fruit stalks were classified into a total of four categories according to the morphological characteristics of fruits at different ripening levels. The categories were as follows: green ripe stage (green), half-ripe stage (half), red ripe stage (red), and fruit stalk (Fig. 2). The half-ripe stage includes the color-changing stage and the pre-red ripe stage, while the red ripe stage includes the mid-red ripe stage and the post-red ripe stage.

**Fig. 3:**
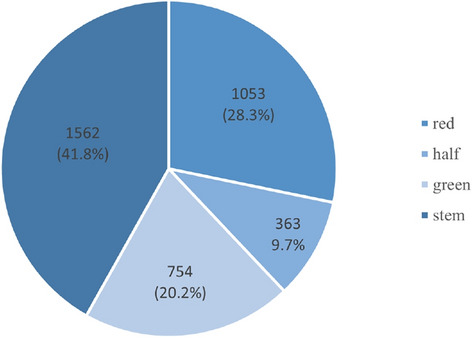
Class label visualization.

A visualization analysis of the annotation files for the training set demonstrates the proportion of tomato class labels for red, half, green, and stem, as illustrated in Fig. 3. It specifically details the number of samples in each of the four categories. The stem class has 1562 samples, green has 754, red has 1053, and half has 363. There is a notable gap in the proportion of stem and half labels, indicating an imbalance in the number of classes. In the actual data collection process, although stems are easily obscured, their number is still much larger than the number of the other three classes, causing an imbalance in the sample classes. This imbalance can lead to a lower accuracy rate for the half class during model training, potentially affecting the overall detection performance of the model.

### YOLOX-SE-GIoU network model

**Fig. 4:**
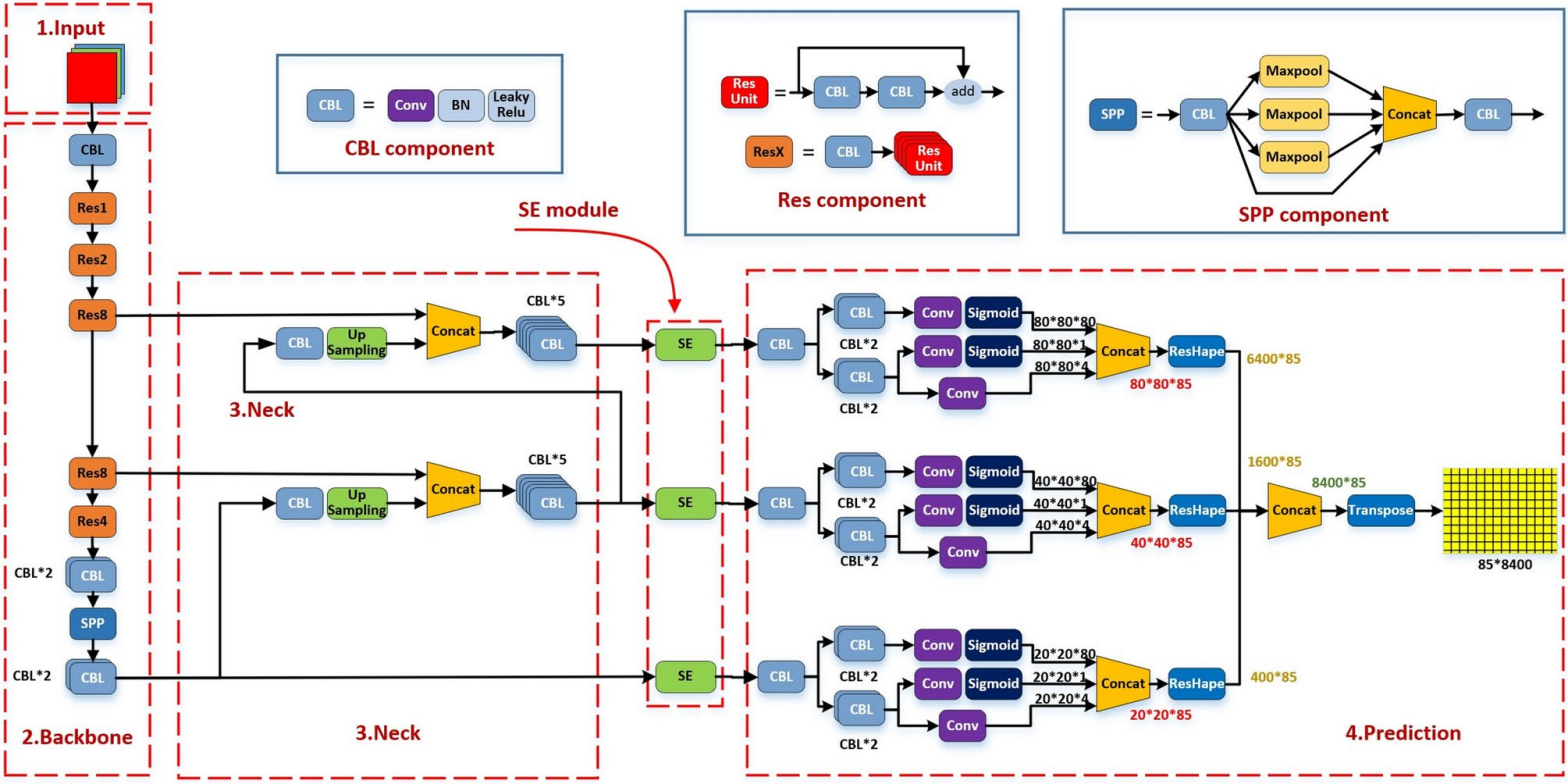
Structure of the YOLOX-SE-GIoU network.

In this experiment, the YOLOX model^24^ is improved and the YOLOX-SE-GIoU model is proposed. The optimized network structure is shown in Fig. 4.

The YOLOX-SE-GIoU network structure consists of four distinct components: Input, Backbone, Neck, and Prediction, as shown in Fig. 4. In the Input layer, an image with a pixel size of 640 × 640 is input, and the data are augmented using two methods: Mosaic and MixUp; the backbone network of the Backbone layer is Darknet53; the Neck layer incorporates the Feature Pyramid Network (FPN) architecture and integrates the SE attention module at the end as this layer’s output; the Prediction layer refines the loss function from the original YOLOX to GIoU loss for improved accuracy.

#### SE attention mechanism

Attention mechanisms are a set of mechanisms that autonomously learn weight coefficients through the network, emphasizing areas of interest and suppressing irrelevant background areas in a “dynamic weighting” manner. The classification of main-stream attention mechanisms currently includes channel attention, spatial attention, hybrid attention, and self-attention mechanisms. Among these, the channel attention mechanism is to obtain the importance of each channel through global pooling of each feature map, and then obtain the weight coefficients that will suppress the unimportant features.

**Fig. 5:**
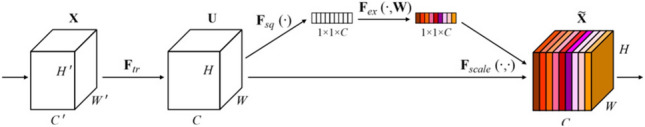
Structure of the SE attention mechanism module.

The SE (Squeeze-and-Excitation) attention mechanism^25^ is a type of channel attention mechanism. This mechanism first performs global average pooling on the input image features. Secondly it passes the image features through two fully connected layers and uses the Sigmoid function to limit the output to between 0-1. Finally, the weights produced by the channel attention mechanism are combined with the original feature maps, resulting in the final feature maps enhanced through the attention mechanism. The architecture of the SE attention mechanism network is illustrated in Fig. 5.

The SE attention mechanism can adaptively select and emphasize important features, improving the discriminative ability of features, better fitting the complexity between channels, and improving the efficiency of processing images.

#### GIoU loss function

In object detection tasks, IoU is used to measure the overlap between the predicted and ground truth bounding boxes. Generalized Intersection over Union (GIoU)^26^ is a loss calculation method for bounding box prediction derived from IoU that is scale-invariant. Compared with IoU Loss optimization part is: adding the influence of non-overlapping regions (in the prediction bounding box and the ground truth box there is no overlap part can also reflect the distance between the two boxes), can better measure to the degree of overlap, has a faster convergence speed. The formula is shown below:

1 $$\begin{aligned} GIoU= & IoU-\frac{{A}^{c}-u}{{A}^{c}} \end{aligned}$$

2 $$\begin{aligned} {L}_{GIoU}= & 1-GIoU \end{aligned}$$

As can be seen from the formula, the final result of calculating GIoU Loss is returned as 1-GIoU. Since the value of 1-GIoU is in the range of [0,2] and has a certain “distance” property, i.e., the larger the overlap area between the predicted bounding box and ground truth box, the smaller the loss, and vice versa, the larger it is; and it can avoid the influence of the target shape size and more accurately reflect the interrelationships of the boxes, and possesses scale invariance.

### Training of network models

#### Test platform and training parameters

The experiments were conducted on a Windows 10 (64-bit) operating system with 16 GB of RAM, graphics card driver GTX 1650Ti, Intel Core i5-10200H CPU @ 2.40GHz processor, programming platform Anaconda 4.12.0, CUDA 10.1, and development environment PyTorch. Programming with Python 3.8.

All models used in this study were trained on the same dataset with consistent hyperparameters to identify tomatoes and their stalks. The models processed images of size 640 × 640 pixels, with a batch size of 16, over 100 epochs. They utilized an Adam optimizer with an initial learning rate of 0.01, a momentum of 0.9, and a weight decay coefficient of 0.005.

In this research, the evaluation metrics commonly used in target detection include Intersection over Union (IoU), Precision, Recall, F1 Score, and mean Average Precision (mAP).

#### Evaluation index of the model

This paper utilizes common target detection evaluation metrics such as Intersection over Union (IoU), Precision, Recall, F1 Score, Precision-Recall (P-R) curves, and mean Average Precision (mAP).

In evaluating the target detection model, IoU is used to quantify the degree of fit, i.e., the quality of detection is judged from the degree of fit between the predicted bounding box and the ground truth bounding box, and its calculation formula is shown in (3):

3 $$\begin{aligned} IoU=\frac{{S}_{A}\bigcap {S}_{B}}{{S}_{A}\bigcup {S}_{B}} \end{aligned}$$

${S}_{A}$ denotes the set of pixel points within the predicted bounding box, while ${S}_{B}$ represents the set of pixel points within the ground truth bounding box.

Precision and Recall assess the performance of information retrieval systems, gauging their effectiveness in identifying relevant items. Precision denotes the detection precision, which represents what proportion of the targets detected by the model are ground truth target objects, and Recall denotes the detection recall, which represents what proportion of all ground truth targets are detected by the model. The Precision and Recall formulas are shown in (4), (5) are shown:

4 $$\begin{aligned} Precision= & \frac{TP}{TP+FP} \end{aligned}$$

5 $$\begin{aligned} Recall= & \frac{TP}{TP+FN} \end{aligned}$$

where TP (True Positive) denotes the number of samples where the model predicts a target box (IoU greater than the threshold) and the category label agrees with the ground truth label; FP (False Positive) denotes the number of samples where the model predicts a target box and the category label does not agree with the actual label; and FN (False Negative) denotes the number of samples where the tomato and the fruit stalk are not detected.

The F1 Score, a weighted average of Precision and Recall, serves as an indicator of the model’s robustness; a higher F1 Score suggests better performance. The formula is shown in (6):

6 $$\begin{aligned} F1=2 \times \frac{Precision \times Recall}{Precision+Recall} \end{aligned}$$

Precision measures the model’s ability to correctly identify negative instances, with higher Precision indicating greater discriminatory power against negatives. Recall measures the model’s ability to identify positive instances, with higher Recall indicating enhanced recognition capabilities.

In the graph of P-R curve, Recall was taken the abscissa and Precision was taken as the ordinate. Precision was negatively correlated with Recall. The larger the area (AP value) surrounded by P-R curve was, the better the model effect was. The mAP value quantifies the model’s average precision across different categories, serving as a benchmark for evaluating its overall target detection proficiency, as detailed in equations (7) and (8):

7 $$\begin{aligned} AP= & \int _{0}^{1}p\left( r\right) dr \end{aligned}$$

8 $$\begin{aligned} mAP= & \frac{\textstyle \sum _{i=1}^{N}A{P}_{i}}{N} \end{aligned}$$

where $p$ denotes Precision and $r$ denotes Recall. AP denotes the average detection accuracy of a single category of the model and $N$ denotes the number of categories.

## Results

### P-R curve analysis

**Fig. 6:**
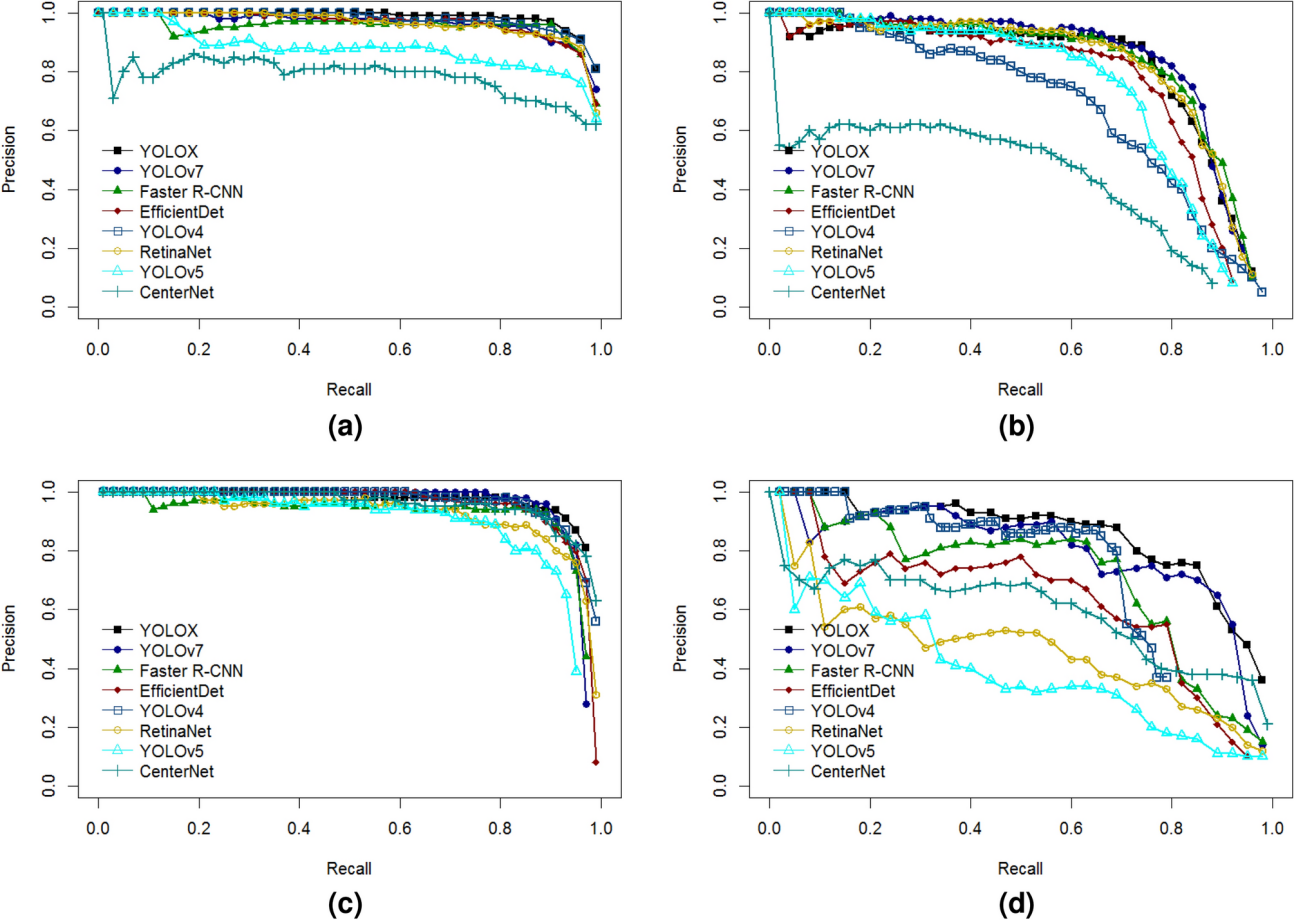
P-R curves for different models on each class. (**a**) P-R curve for the “red” category; (**b**) P-R curve for the “half” category; (**c**) P-R curve for the “green” category; (**d**) P-R curve for the “stem” category.

In order to compare the prediction of CenterNet, RetinaNet, EfficientDet, Faster R-CNN, YOLOv4, YOLOv5, YOLOv7, and YOLOX models on tomato ripeness and fruit peduncle in the P-R curves, the predictions of the P-R curves predicted by the above models for the test set (in this paper, a total of 200 images in the test set are used, comprising 149 green, 62 half, 231 red, and 30 stem classes) are plotted separately. The larger the area under the line of the P-R curve, the better the model effect. The results are shown in Fig. 6 below.

For the P-R curves analysis of the red ripe tomato category (red), it can be seen that, except for YOLOv5, CenterNet and Faster R-CNN models, all the other models are located above the coordinates. Although the difference is small, before the Recall value of 0.9, the YOLOX model curves are occupying the uppermost position, so the area under the line of its curves is larger than the other models that is more applicable to the category.

The analysis of the P-R curve for the half-ripe tomato category (half) shows that the YOLOX, YOLOv7 and YOLOv4 models have overlapping P-R curves before the Recall value of 0.4, but after the Recall value of 0.4, the curves of the YOLOv7 and YOLOv4 models begin to gradually decline, while the YOLOX model still occupies the uppermost position. A comprehensive analysis shows that the YOLOX model has a larger area below the curve line and is more suitable for this category.

The analysis of the P-R curve for the green ripe tomato category (green) shows that, except for Faster R-CNN, RetinaNet and YOLOv5, all the other curves are closer to the upper right of the axis and the gap is smaller. However, when the Recall value is 0.9, the YOLOX model is more suitable for this data category because the curve of YOLOX model decreases slower than the other models and the curve position is closer to the top, so the area under the line is slightly larger than the other models.

The analysis of the P-R curve graph for the tomato fruit stalk category (stem) shows that, except for the CenterNet model, the Precision value of each model decreases as the Recall value increases, with the relatively higher curves of the Faster R-CNN, RetinaNet and YOLOX models. So, for this category, the overall gap between the above three models is small and all of them can be used to recognize this category.

Combining the above P-R curve analysis of each tomato ripeness and fruit stalk category, the YOLOX model has a better overall effect in multi-category recognition compared to the other models, and is more suitable for the detection and recognition of tomato ripeness and fruit stalk dataset.

### Comparison of recognition results of YOLO series models

To assess the recognition performance of the YOLO series of models on the tomato dataset, this study employs four distinct network models, specifically YOLOv4, YOLOv5, YOLOv7, and YOLOX, to train the aforementioned training set comprising 640 images. The resulting experimental comparison outcomes are summarized in Table 2.

**Table 2:** YOLO recognition results of different models in the same series.

Model	F1/%	mAP/%	AP/%			
			Red	Half	Green	Stem
YOLOv4	60.05	82.88	97.57	68.43	96.19	69.33
YOLOv5	54.21	69.96	79.69	60.66	95.68	43.82
YOLOv7	81.37	85.59	93.46	82.35	98.93	67.62
YOLOX	85.25	91.00	97.50	85.64	95.81	85.07

The comparison results in Table 2 show that the mAP value of YOLOX model on the tomato dataset is 8.12%, 21.04%, and 5.41% higher than that of YOLOv4, YOLOv5, and YOLOv7 models, respectively, and the YOLOX model on the “half” unbalanced class is 17.21, 24.98, and 3.29% higher than that of the other models, respectively, and the YOLOX model has the highest F1 value of 85.25. The YOLOX model integrates several advanced mechanisms that distinguish it from other model series. YOLOX adopts an anchor-free design, simplifying the model architecture and reducing computational complexity, thereby enhancing the model’s flexibility in handling objects of varying scales and aspect ratios. The decoupled head design improves the model’s ability to accurately classify and locate objects. Additionally, the integration of SimOTA (Simplified Optimal Transport Assignment) further optimizes the matching strategy between predicted boxes and ground truth, thereby improving training efficiency and detection performance. It can be seen that after applying the aforementioned three mechanisms, YOLOX model has significantly improved the detection rate and accelerated the model convergence, which is more stable than the other models as a whole. Therefore, in this paper, the YOLOX model is chosen as the main algorithm model for this experiment, and on this basis, it is improved to enhance the accuracy of tomato different ripeness and fruit stalks identification.

### Comparison results of YOLOX model optimization

#### Results and analysis of experiments on the mechanism of adding attention

**Fig. 7:**
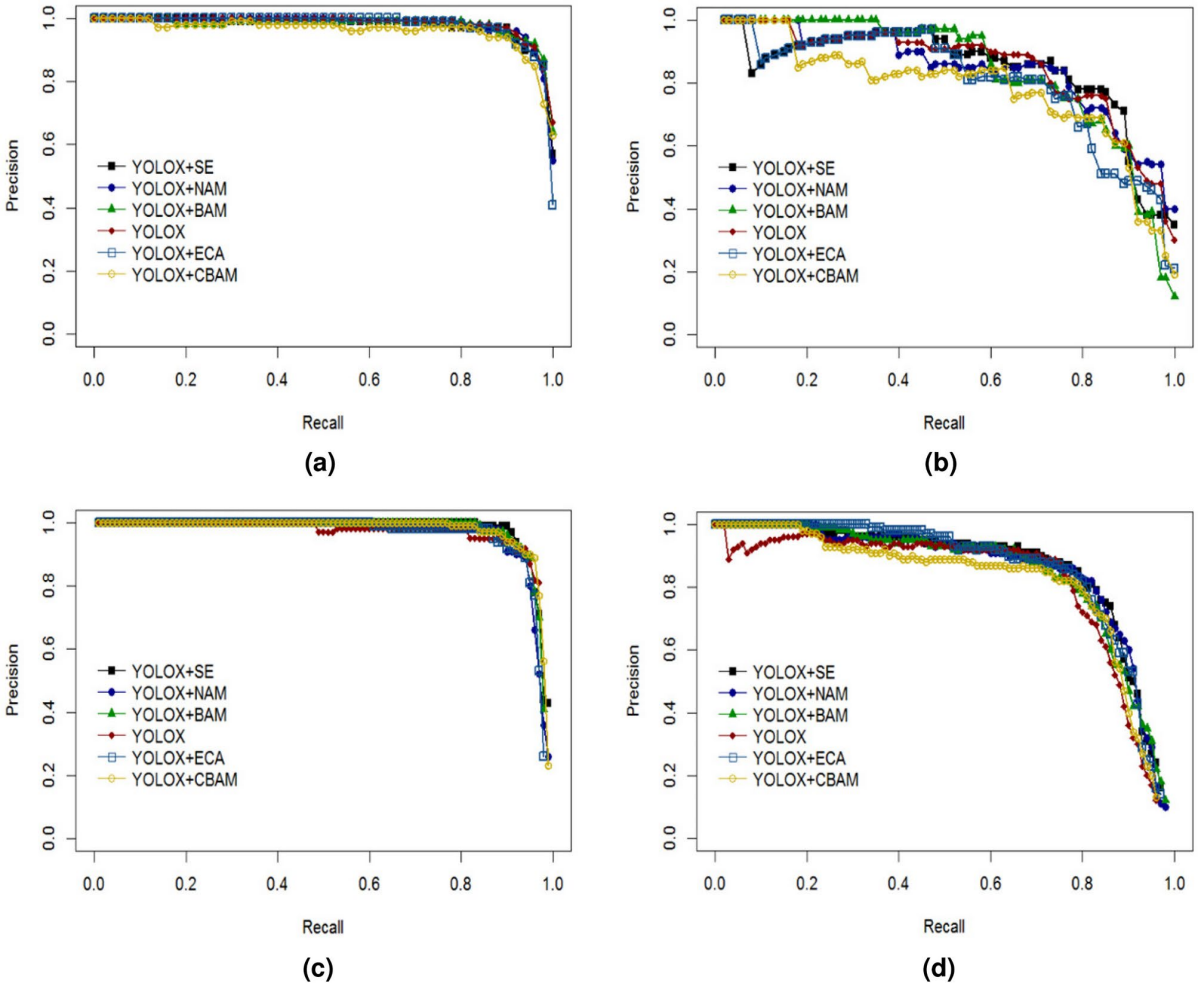
P-R curves of YOLOX models with different attention modules on each class. (**a**) P-R curve for the “red” category; (**b**) P-R curve for the “half” category; (**c**) P-R curve for the “green” category; (**d**) P-R curve for the “stem” category.

In order to improve the model’s accurate recognition rate of unbalanced samples, five attention modules, SE, ECA, BAM, CBAM, and NAM, were added to the end of the neck network of YOLOX model respectively for experiment, using the test set of 200 images, to validate the effect of different attention mechanisms on the recognition accuracy of the model. The comparison of the model P-R curves after adding the attention modules is shown in Fig. 7 below.

The analysis of the P-R curve graphs for the red ripe tomato category (red) shows that the curves of the models are located in the upper right of the coordinates, with a smaller gap, except for the YOLOX+CBAM model, which is more obviously below the other models, but the YOLOX+SE model’s P-R curves occupy the uppermost position until the Recall value of 0.0-0.6, so that the area under the line of the curve of the YOLOX+SE model is larger than that of the other models and more applicable to the category.

The analysis of the P-R curve for the half-ripe tomato category (half) shows that before Recall values of 0.25, although the P-R curves of YOLOX+BAM are higher than those of other models, after Recall value of 0.4, the curves of the YOLOX+SE model are progressively higher than those of YOLOX+BAM and the curves of YOLOX+BAM drops faster than that of YOLOX+SE model. The comprehensive analysis shows that the curve of YOLOX+SE model has a larger area below the line and is more suitable for this category.

The analysis of the P-R curve for the green ripe tomato category (green) shows that all the curves are located directly above the axes until the Recall value of 0.8, except for the YOLOX curve, which is significantly lower than the other models after the Recall value of 0.4. At Recall value of 0.9, except for the YOLOX+SE model curve which is still located at the top, all the other curves are decreasing to different degrees, and the YOLOX+SE model decreases slower than the other model curves, so the area under the line is slightly larger than the other models, so it is more applicable to this data category.

For the tomato fruit stalk category (stem) the analysis of the P-R curve shows that all the curves except YOLOX, YOLOX+CBAM and YOLOX+BAM are clearly located in the coordinates directly above and almost overlap with a small gap. So, for this category, the overall gap between the three models YOLOX+SE, YOLOX+NAM and YOLOX+ECA is small, and all of them can be used to identify this category.

Combining the above P-R curve analyses for each tomato ripeness and stalk category, the gap between each curve is smaller in the red, green and stem categories, which is due to the larger proportion of the data itself and the clearer target characteristics. However, in the half category with a smaller number of samples, the advantage of the YOLOX+SE model is more obvious, so the YOLOX+SE model has a better integrated effect in multi-category identification compared to other models, and is more suitable for the detection and identification of tomato ripeness and fruit stem datasets.

#### Attention module comparison results

To assess the impact of various attention mechanisms on model recognition accuracy, five attention modules–SE, ECA, BAM, CBAM, and NAM–were integrated at the end of the neck network of the YOLOX model for experimentation. The results are displayed in Table 3:

**Table 3:** Comparison of YOLOX model results after adding attention module.

Model	Precision/%	Recall/%	F1/%	mAP/%	AP/%			
					Red	Half	Green	Stem
YOLOX	83.39	87.51	85.25	91.00	97.50	85.64	95.81	85.07
YOLOX-BAM	82.86	86.63	84.50	90.59	98.12	83.88	96.29	84.09
YOLOX-CBAM	80.97	86.11	83.25	88.06	95.95	78.46	96.72	81.13
YOLOX-ECA	83.90	85.90	85.00	89.96	97.65	81.66	95.28	85.24
YOLOX-NAM	82.38	87.62	85.00	90.92	97.82	84.87	95.65	85.32
YOLOX-SE	86.36	88.33	86.00	91.92	97.99	88.03	94.91	86.76

The YOLOX model enhanced with the SE attention module shows an increase in mean Average Precision (mAP) by 0.92% over the original model, with an F1 score of 86.00%. Additionally, the Precision and Recall have improved by 2.97% and 0.82%, respectively, compared to the original model. It indicates that the SE attention module enhances the half category precision and also strengthens the relationship between the features, so that the overall precision of the model is improved. Conversely, the inclusion of BAM, NAM, CBAM, and ECA attention modules resulted in a decrease in mean average precision by 0.41%, 0.08%, 2.94%, and 1.04%, respectively, relative to the original model. Other performance metrics also declined to varying extents. This suggests that spatial attention mechanisms, while adding more parameters, might overlook crucial information of specific categories within complex images, thereby impairing the model’s performance.

The analysis of individual category average precision (AP) from Table 3 reveals that the SE attention module enhances the model’s focus on useful channel information by learning adaptive channel weights. This adjustment allows the model to better capture the complex interrelations between channels, improving performance across all four classes samples. Notably, the detection and identification precision for the imbalanced sample reached 88.03%, marking an improvement of 2.38% over the original model. With the addition of the SE attention module, the YOLOX model’s accuracy and stability for imbalanced samples are significantly enhanced, making it more effective in identifying varying ripeness levels and stalks of tomatoes.

#### Experimental results and analysis of loss function optimization

**Fig. 8:**
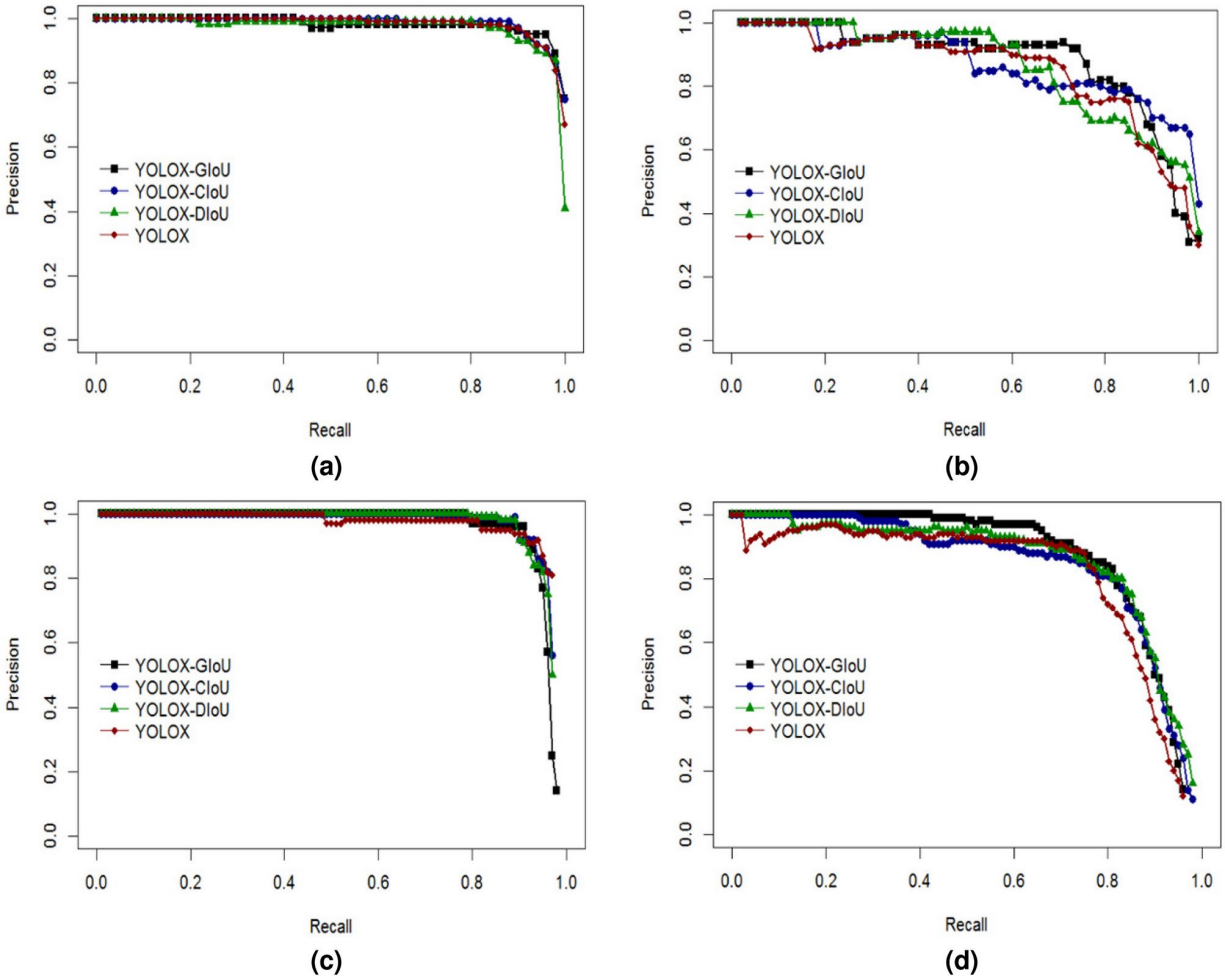
P-R curves of YOLOX models with different loss functions on each class. (**a**) P-R curve for the “red” category; (**b**) P-R curve for the “half” category; (**c**) P-R curve for the “green” category; (**d**) P-R curve for the “stem” category.

In order to select the most suitable loss function for this paper’s dataset and improve the robustness and overall detection accuracy of the model, this experiment re-places and compares the three loss functions of DIoU, CIoU and GIoU loss with the IoU loss in YOLOX, using the test set of 200 images, and the comparison of the model’s P-R curves after replacing the loss functions is shown in Fig. 8 below.

The analysis of the P-R curve for the red ripe tomato category (red) shows that the YOLOX model curve is clearly located below the other models and the gap between the rest of the models is smaller. After the Recall value of 0.9, the YOLOX-GIoU model curve occupies the uppermost position, so its area under the curve line is larger than the other models and is more applicable to this category.

The analysis of the P-R curve graphs for the half-ripe tomato category (half) shows that the models show different degrees of decline after a Recall value of 0.2, and the P-R curves of the models remain coincident. However, at the Recall value of 0.5, there is a gap between the model curves. From the Recall value of 0.6 to 0.85, the YOLOX-GIoU model always keeps the top position. Comprehensively analyzing the whole curve, it can be seen that the YOLOX-GIoU model’s curve maintains the uppermost position more than the other models, so it has a larger area under the line, which is more suitable for this category.

The analysis of the P-R curve for the green ripe tomato category (green) shows that at Recall values of 0-0.5, the P-R curves of the models remain coincident, after which the YOLOX model decreases compared to the other models. At Recall value of 0.9, the YOLOX-GIoU model remains at the top and all other models start to decrease. So YOLOX-GIoU model is more suitable for this category.

For the P-R curve analysis of the tomato fruit stalk category (stem), it can be seen that the Precision value of each model is decreasing with the increase of Recall value, in which the YOLOX-GIoU model curve is relatively higher than the other models, and the rate of decrease is more moderate. So, for this category, YOLOX-GIoU model is more applicable.

Combining the above P-R curve analyses for each tomato ripeness and fruit stalk category, the YOLOX-GIoU model exhibits superior multi-category recognition capabilities, making it particularly well-suited for detecting and recognizing varying stages of tomato ripeness and fruit stalks.

#### Loss function comparison results

To identify the most effective loss function for this study’s dataset and to enhance the model’s robustness and overall detection accuracy, this experiment compared DIoU loss, CIoU loss, and GIoU loss against the standard IoU loss in YOLOX. The comparative results are displayed in Table 4:

**Table 4:** Comparison of detection results after replacing the loss function with YOLOX.

Model	Precision/%	Recall/%	F1/%	mAP/%	AP/%			
					Red	Half	Green	Stem
YOLOX	83.39	87.51	85.25	91.00	97.50	85.64	95.81	85.07
YOLOX-DIoU	81.53	85.16	83.25	91.07	97.36	85.37	95.85	85.67
YOLOX-CIoU	84.51	86.06	86.00	91.35	97.59	86.22	96.26	85.33
YOLOX-GIoU	84.72	88.11	86.25	91.44	97.33	86.65	95.61	86.18

As can be seen from Table 4, under the same conditions, the use of the GIoU loss function improves the mAP value by 0.44% and the F1 value by 1.00% with respect to the original model. It shows that in this experimental dataset, GIoU loss can better reflect the model’s overlap between the predicted box and the ground truth box, while also focusing on the differences between fruits and fruit stalks. This improves the recognition accuracy of each category and increases the overall stability of the model. Replacing the loss function with DIoU loss and CIoU loss also has some improvement over the original model, which are 0.07% and 0.35%, respectively, where DIoU loss is lower than the original model by 1.86%, 2.35%, and 2.00% in terms of model detection accuracy, recall, and F1 value, respectively, which indicates that after using DIoU loss and CIoU loss, the convergence speed of the regression process is faster, but leads to the model recognition performance decreases. CIoU loss has an improvement over the DIoU loss after considering the aspect ratio of the bounding box, but the overall performance is lower than that of GIoU loss.

In summary, when replacing the loss function of the model with GIoU loss com-pared to the original YOLOX model, the problem of low recognition accuracy caused by the large difference in the target scales of the fruit and the fruit stalk is improved, indicating that this loss function is more applicable to the tomato dataset of this experiment.

#### Ablation experiment

To reflect the effect of adding both SE Attention Module and GIoU loss on the performance of the YOLOX model, comparative validation was performed using ablation experiments on the test set of 200 images.

**Fig. 9:**
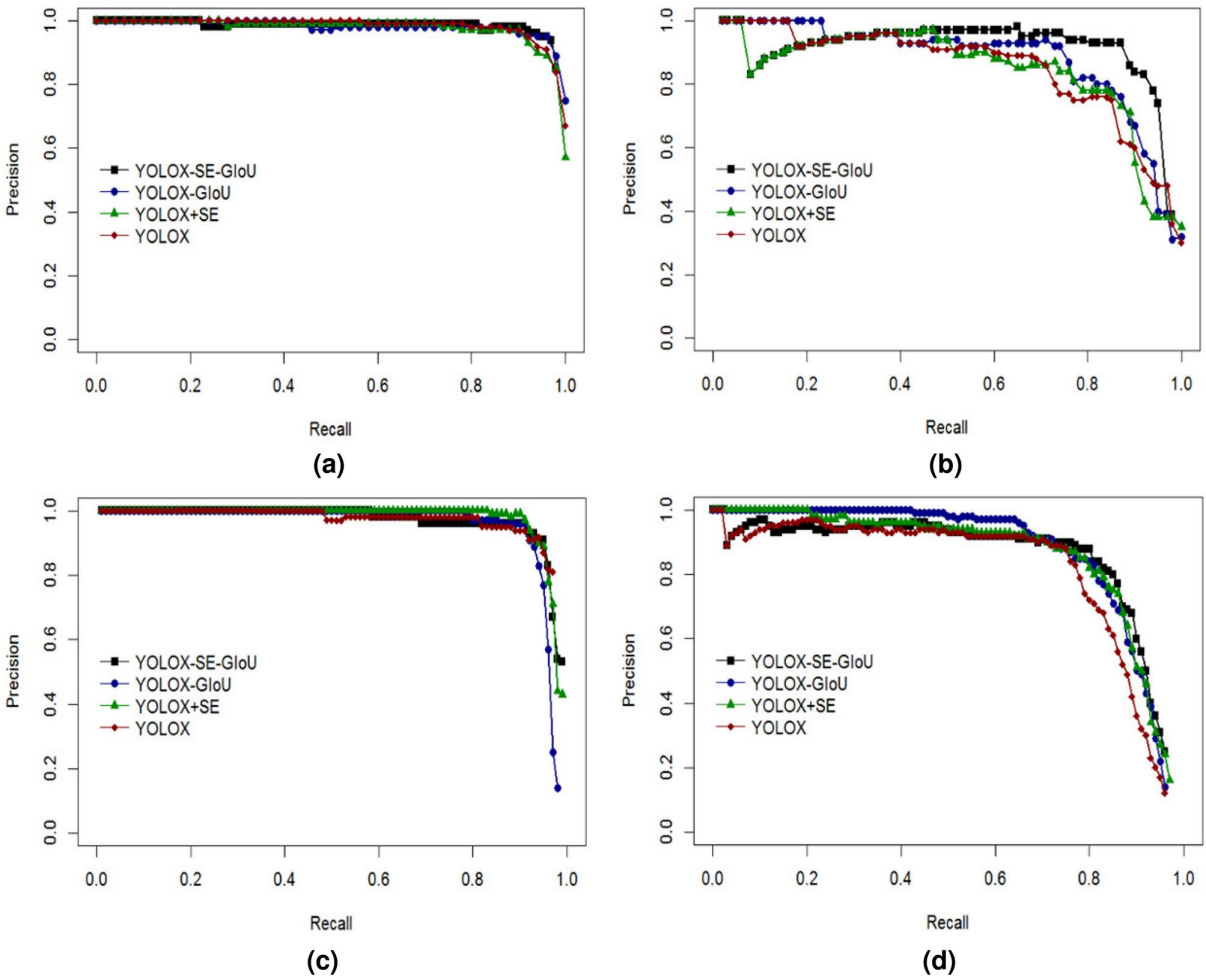
P-R curves of all models in each category under ablation experiment. (**a**) P-R curve for the “red” category; (**b**) P-R curve for the “half” category; (**c**) P-R curve for the “green” category; (**d**) P-R curve for the “stem” category.

A comparison of the model P-R curves is shown in Fig. 9 below.

The analysis of the P-R curve for the red ripe tomato category (red) shows that all model curves are located above the coordinates and the gap is small, but after the Recall value of 0.8, the YOLOX-SE-GIoU model curves all occupy the uppermost position, so that the area under the line of their curves is larger than that of the other models, which is more applicable to this category.

The analysis of the P-R curve for the half-ripe tomato category (half) shows that before the Recall value of 0.2, the YOLOX-GIoU model is located at the top compared to the other models. After the Recall value of 0.2, the YOLOX-SE-GIoU model’s curve is always located above the other models, and a comprehensive analysis shows that the YOLOX-SE-GIoU model has a larger area under the curve line, which is more suitable for this category.

The analysis of the P-R curve for the green ripe tomato category (green) shows that the YOLOX model curve is clearly lower than that of other models, with the smallest area below the line. Throughout the later stages of the curve, the YOLOX-SE-GIoU model, on the other hand, declines more slowly than the other model curves and contains a larger range. It is therefore more applicable to this data category.

The analysis of the P-R curve for the tomato fruit stalk category (stem) shows that the YOLOX-GIoU and YOLOX+SE model curves are located at the top of the coordinates until the Recall value of 0.2. Between Recall values of 0.2-0.7, the YOLOX-GIoU model curve is located at the uppermost coordinate. However, after the Recall value of 0.7, the YOLOX-SE-GIoU model P-R curve is higher than the other models and decreases more slowly than the other models. So, for this category, although the gap between the above models is small in the early stage, the curves all start to decrease in the later stage with the increase of Precision value, and the improved YOLOX-SE-GIoU model decreases more slowly, which indicates that the improved model is more stable and has better performance.

Combined with the above analysis of P-R curves for each tomato ripeness and fruit stalk category, the improved YOLOX-SE-GIoU model has a better overall effect in multi-category recognition compared to other models, and is more suitable for the detection and recognition of tomato ripeness and fruit stalk datasets.

#### Comparison of ablation performance of YOLOX model

To reflect the combined effect of adding the SE attention module and GIoU loss on model performance, an ablation study was conducted for comparative validation. The experimental results are shown in Table 5.

**Table 5:** Results of model ablation experiments.

Model	SE	GIoU	Precision/%	Recall/%	F1/%	mAP/%	AP/%			
							Red	Half	Green	Stem
YOLOX			83.39	87.51	85.25	91.00	97.50	85.64	95.81	85.07
YOLOX①	$\checkmark$		86.36	88.33	86.00	91.92	97.99	88.03	94.91	86.76
YOLOX②		$\checkmark$	84.72	88.11	86.25	91.44	97.33	86.65	95.61	86.18
YOLOX③	$\checkmark$	$\checkmark$	87.07	87.74	87.25	92.17	97.01	87.52	95.30	88.85

As can be seen from Table 5, adding the SE attention module enhances the model’s ability to focus on salient features specific to tomatoes, such as color variations and shape details, by adaptively recalibrating channel-wise feature responses. This improves feature discriminability and results in a mAP increase of 0.92%. In this study, the SE attention module adaptively recalibrates channel-wise feature responses, enhancing the model’s focus on significant characteristics of tomatoes, including color variations and shape details, thereby improving feature discrimination and leading to a mAP increase of 0.92%. In order to obtain a more accurate prediction box and reduce the problem of accuracy degradation caused by different target scales, the loss function in YOLOX is replaced with GIoU loss function, which provides a better measure of the overlap between predicted and ground truth boxes, especially for small and elongated structures like tomato stems. This leads to a mAP improvement of 0.44%. GIoU loss offers a more comprehensive evaluation of the overlap between predicted and ground truth bounding boxes, particularly improving accuracy in cases involving small and elongated structures such as tomato stems, thereby enhancing the mAP by 0.44%. In order to improve the overall performance and stability of the model, the two optimization strategies are fused, and the final optimized YOLOX-SE-GIoU model benefits from both the enhanced feature discrimination for tomato characteristics and more accurate bounding box regression for stems, improving the APs of the unbalanced sample “half” and the small-scale target “stem” by 1.88% and 3.78%, respectively, compared with the original model; the mAP and the F1 value of the single optimization strategy are also improved to a certain extent, which effectively reduces the phenomena of miss detection and false detection in tomato identification. In addition, we evaluated the inference speed using the FPS metric, and the FPS of the YOLOX-SE-GIoU model is about 45, which basically meets the real-time processing requirements of embedded harvesting devices.

In summary, YOLOX-SE-GIoU was confirmed as the final model for this experiment, and it was applied to the tomato test set to verify the effect.

### Model detection effectiveness analysis

**Fig. 10:**
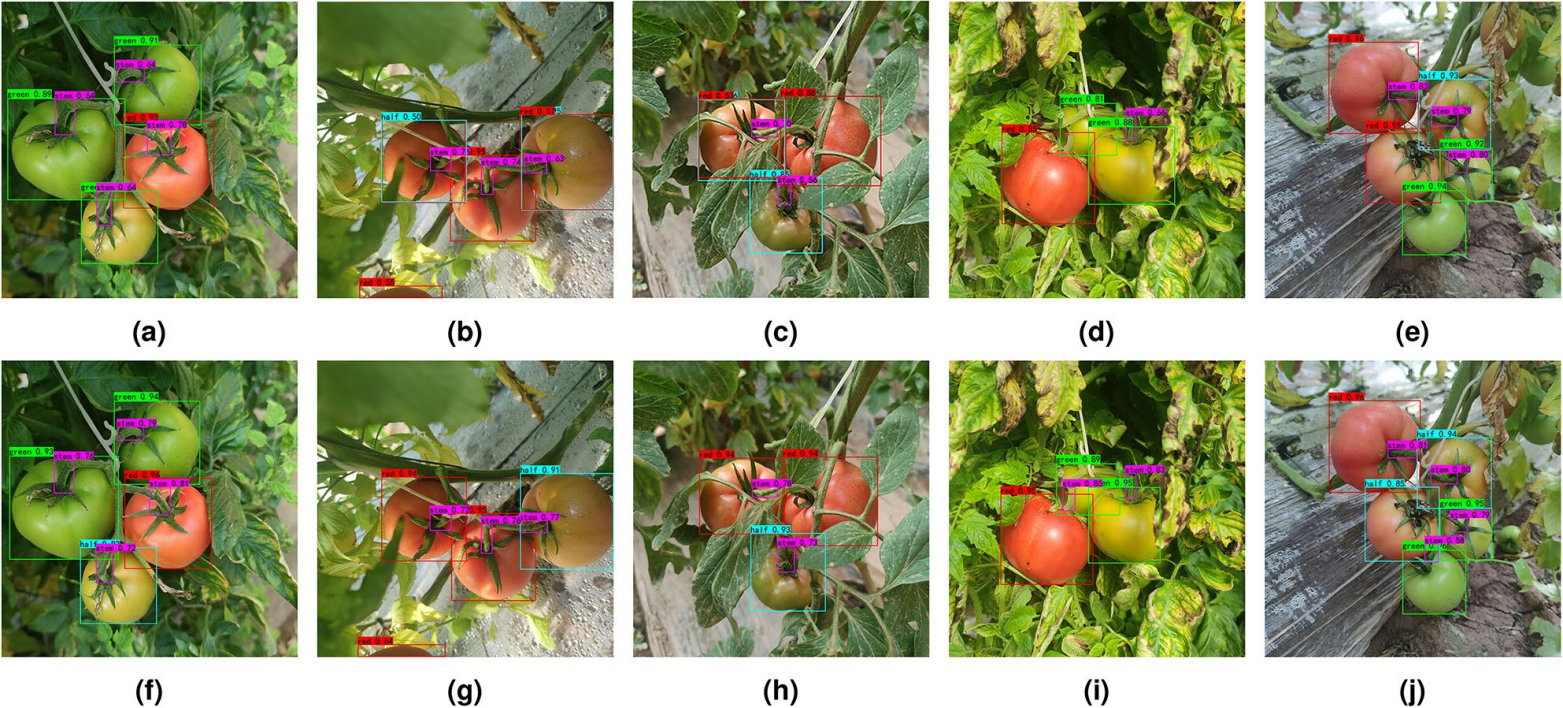
Comparison of visual results of YOLOX-SE-GIoU(**f**–**j**) and YOLOX(**a**–**e**) tomato fruit and stem detection.

A comparison of the testing effect of YOLOX-SE-GIoU model on the test set of 200 images is shown in Fig. 10. From the figure, it can be seen that under direct lighting, a half-ripe tomato is misclassified as a green ripe tomato in Fig. 10a; under backlighting, a red ripe tomato is misclassified as a half-ripe tomato in Fig. 10b, while a half-ripe tomato is misclassified as both a red ripe and a half-ripe tomato; due to occlusion from leaves, a red ripe tomato is misclassified as both half-ripe and red ripe in Fig. 10c; due to occlusion by an object, the fruit stalk of a green ripe tomato is missed in Fig. 10d. Although the improved model fails to detect this fruit stalk, its overall confidence level is higher than that of the original YOLOX model. Additionally, the fruit stalk of a red ripe tomato is not recognized due to its color similarity with the green ripe tomato. The short fruit stalk of a green ripe tomato is missed due to occlusion by leaves, and the half-ripe tomato is misclassified as a red ripe tomato in Fig. 10e. Figure 10i further shows that the improved model still experiences some missed detections in cases of partial occlusion and overlap, an area for future improvement. In conclusion, as shown in Fig. 10f–h,j, the YOLOX-SE-GIoU model is not affected by occlusion, lighting, distance, and other factors, and can accurately identify tomatoes at different ripeness levels and their fruit stalks, with an improved confidence level compared to the original model.

## Discussion

The enhanced performance of the YOLOX-SE-GIoU model in recognizing tomato ripeness and stems, as reported in the results section, underscores the effectiveness of integrating the SE attention module and GIoU loss function into the YOLOX framework. The attention mechanism’s focus on salient features significantly improved the precision and recall rates across various stages of tomato maturity, facilitating a reduction in both false positives and false negatives. This precision is crucial for automated harvesting robots, where accuracy directly impacts the quality and quantity of yield.

The GIoU loss function specifically addressed the challenges posed by tomatoes and stems of varying sizes and overlapping instances. By measuring the shapes and orientations of predicted and actual bounding boxes more comprehensively, the model could more accurately segment and classify each component, which is vital for precise cutting and handling by robotic systems.

However, the limitations in the experimental setup, such as dataset diversity and environmental control, suggest areas for future improvement. While the model performs well under controlled conditions, its robustness in natural and varied agricultural fields remains to be tested extensively. Future work could also explore the scalability of the proposed model enhancements in larger, more heterogeneous datasets to further validate and refine its effectiveness.

Continued research is needed to explore the integration of additional sensory data, such as depth and thermal imaging, to complement the visual recognition capabilities of the model. This could potentially enhance the system’s ability to operate under diverse environmental conditions, including varying lighting and weather scenarios.

## Conclusion

In this experiment, to address the problem of poor recognition of tomatoes due to occlusion, overlap, sample imbalance and large differences in the scale of fruit stalks, we establish a diverse tomato dataset, add the SE attention module and replace the loss function with GIoU loss in the YOLOX model to improve the model’s accurate recognition of tomato with different ripeness and fruit stalks, and compare it with other models to draw the following conclusions:

1. On the tomato dataset of this experiment, the mAP of the YOLOX-SE-GIoU model reaches 92.17%. Compared to YOLOv4 (82.88%), YOLOv5 (69.96%), YOLOv7 (85.59%), and YOLOX (91.00%), YOLOX-SE-GIoU exhibits improvements of 9.29%, 22.21%, 6.58%, and 1.17%, respectively. The AP of the imbalanced sample “half” is improved by 1.68-26.66%, the AP of the smaller scale “stem” is improved by 3.78–45.03%, and the F1 value is improved by 2.00-33.04%. The combined identification performance of the YOLOX-SE-GIoU model for imbalanced and different scale samples are superior to that of the original model and other models of the same series.
2. Incorporating the SE attention module enhances target detection accuracy against complex backgrounds, particularly improving the recognition accuracy of imbalanced samples such as “half”. This adjustment significantly reduces missed and false detections during the tomato picking process, thereby elevating overall model recognition accuracy. Additionally, replacing the loss function with the GIoU loss function can better reflect the overlap between the predicted box and the ground truth box, which effectively improves the recognition accuracy of the stem category at the small scale, and overall, the model demonstrates improved accuracy and stability compared to other loss functions.
3. In practical robotic harvesting scenarios, hardware constraints such as limited computational power, energy consumption, and device may affect the real-time performance and deployment of the proposed model. Future research could focus on further optimizing the model’s complexity and algorithmic efficiency to ensure seamless integration with robotic harvesting systems, as well as exploring hardware acceleration and model compression techniques to mitigate these limitations, ultimately aiming for stable and efficient operations in agricultural production environments.

In this study, the proposed YOLOX-SE-GIoU model, which integrates the SE attention module and the GIoU loss function, enhances the accuracy and stability of tomato ripeness recognition. It effectively detects both the ripeness of tomatoes and the classification of fruit stalks, thereby reducing missed detections and false positives during the harvesting process, which further alleviates the workload of farmers. However, these conclusions are based on specific experimental conditions, and factors such as occlusion and lighting variations in real agricultural environments may impact the model’s performance. Therefore, future research will focus on optimizing the model’s complexity and algorithmic efficiency to address the challenges associated with integrating robotic harvesting systems, ultimately ensuring the model’s effectiveness and reliability in actual tomato production and harvesting processes.

## Data Availability

Access to these data was granted under specific permissions for this research project, and therefore they cannot be shared publicly. However, the data are available from the corresponding author upon reasonable request.
